# Validation of the M. D. Anderson Symptom Inventory multiple myeloma module

**DOI:** 10.1186/1756-8722-6-13

**Published:** 2013-02-05

**Authors:** Desiree Jones, Elisabeth G Vichaya, Xin Shelley Wang, Loretta A Williams, Nina D Shah, Sheeba K Thomas, Valen E Johnson, Richard E Champlin, Charles S Cleeland, Tito R Mendoza

**Affiliations:** 1 Department of Symptom Research, The University of Texas MD Anderson Cancer Center, 1515 Holcombe Boulevard, Unit 1450, 77030, Houston, TX, USA; 2 Department of Stem Cell Transplantation and Cellular Therapy, The University of Texas MD Anderson Cancer Center, 1515 Holcombe Boulevard, Unit 423, 77030, Houston, TX, USA; 3 Department of Lymphoma/Myeloma, The University of Texas MD Anderson Cancer Center, 1515 Holcombe Boulevard, Unit 429, 77030, Houston, TX, USA; 4 Department of Biostatistics, The University of Texas MD Anderson Cancer Center, 1515 Holcombe Boulevard, Unit 1411, 77030, Houston, TX, USA; 5 Current address: Department of Statistics, Texas A&M University, 525K Blocker, 3143 TAMU, College Station, 77843-3143, Houston, TX, USA

**Keywords:** Symptoms, Assessment, Validation, Multiple myeloma, MDASI

## Abstract

**Background:**

The symptom burden associated with multiple myeloma (MM) is often severe. Presently, no instrument comprehensively assesses disease-related and treatment-related symptoms in patients with MM. We sought to validate a module of the M. D. Anderson Symptom Inventory (MDASI) developed specifically for patients with MM (MDASI-MM).

**Methods:**

The MDASI-MM was developed with clinician input, cognitive debriefing, and literature review, and administered to 132 patients undergoing induction chemotherapy or stem cell transplantation. We demonstrated the MDASI-MM’s reliability (Cronbach α values); criterion validity (item and subscale correlations between the MDASI-MM and the European Organization for Research and Treatment of Cancer Quality of Life Questionnaire (EORTC QLQ-C30) and the EORTC MM module (QLQ-MY20)), and construct validity (differences between groups by performance status). Ratings from transplant patients were examined to demonstrate the MDASI-MM’s sensitivity in detecting the acute worsening of symptoms post-transplantation.

**Results:**

The MDASI-MM demonstrated excellent correlations with subscales of the 2 EORTC instruments, strong ability to distinguish clinically different patient groups, high sensitivity in detecting change in patients’ performance status, and high reliability. Cognitive debriefing confirmed that the MDASI-MM encompasses the breadth of symptoms relevant to patients with MM.

**Conclusion:**

The MDASI-MM is a valid, reliable, comprehensive-yet-concise tool that is recommended as a uniform symptom assessment instrument for patients with MM.

## Background

Notwithstanding the use of novel agents [1] and treatment advances such as autologous hematopoietic stem cell transplantation (HSCT), multiple myeloma (MM) is often associated with significant disease-related and/or treatment-related side-effects [2-5]. Because toxicities from induction therapy and HSCT are common [2-4], the symptom burden associated with MM or its treatment (eg, bone pain, fatigue, muscle weakness, nausea, vomiting, constipation, diarrhea) can be severe. Thus, effective symptom management becomes a critical component of patient care.

Accurate symptom assessment in patients with MM requires psychometrically validated tools that are clinically efficient to administer. Instruments used previously (eg, the European Organization for Research and Treatment of Cancer (EORTC) Quality of Life Questionnaire (QLQ-C30) [6] and the EORTC Multiple Myeloma Module (QLQ-MY20)) [7] include some symptom items but primarily assess health-related quality of life (QOL). Further, the QLQ-MY20 is administered in tandem with the QLQ-C30 [7], resulting in a 50-item measure that can create substantial patient burden. Other QOL instruments (eg, the Functional Assessment of Cancer Therapy – Multiple Myeloma) and scales assessing general cancer-related symptoms (eg, the M. D. Anderson Symptom Inventory (MDASI)) [8] have been used in patients with MM [9,10]. However, they do not capture the breadth of symptoms unique to MM or its treatment.

To address this need, we developed a MDASI module specifically for patients with MM. The MDASI is a reliable, valid instrument that was designed for ease of administration and that asks patients to rate 13 symptoms (known as the “core” items) and 6 symptom-related interference items that are common across cancer types and treatments [8]. A recent systematic review of symptom-assessment instruments identified several advantages of the MDASI compared with other measures [11]. The MDASI is comprehensive yet brief, thus minimizing patient burden, and has a readily understood numeric scale that can be adapted for telephone, electronic, or other digital forms of administration. The developers of the original MDASI recognized that particular cancer types or treatments may require the addition of specific symptom items to the MDASI to ensure a comprehensive symptom assessment [12]. These MDASI “modules” include the original MDASI’s 13 core symptom and interference items augmented by additional symptom items specific to certain cancers or treatments. MDASI modules have been developed and validated for patients with a variety of cancers [12-15]. This study describes the psychometric properties and validation of a MDASI module for MM (MDASI-MM).

## Methods

### Participants

Data were derived from 2 studies in patients with MM treated at The University of Texas MD Anderson Cancer Center in Houston, Texas, between 2008 and 2011. The first study assessed symptoms during induction therapy, and the second, during HSCT. For the induction sample, eligible patients had received ≤2 cycles of chemotherapy and were scheduled for bortezomib or thalidomide. Patients were assessed upon enrollment (baseline) and at the end of induction to capture change in symptom profiles in response to therapy. The transplant sample was derived from patients undergoing autologous HSCT. Patients were assessed before beginning high-dose melphalan treatment (pre-HSCT) and at nadir of treatment (7 days post-HSCT). Patients with relapsed or refractory disease were not excluded from the study.

Additional inpatients [16] with MM were interviewed for cognitive debriefing of the MDASI-MM.

All patients were 18 years of age or older, spoke English, and provided written informed consent. The studies were approved by the MD Anderson Institutional Review Board.

### Data collection

#### Module development and cognitive debriefing

Symptom data was obtained via the MDASI-MM (Additional file 1: Figure S1). The MDASI-MM was developed using methods employed for previous MDASI modules [14,15]. MM-related candidate items were derived through patient report of symptoms to clinicians via informal interviews, through clinician and researcher input, and through an exhaustive literature search that included a review of symptoms associated with both MM and its treatment. The resulting MM-specific symptom items were added to the original MDASI to form a provisional MDASI-MM, which was then used for comprehensive patient interviews (cognitive debriefing) with a sample of 20 inpatients with MM. Cognitive debriefing is a critical component of instrument development during which patients assess the relevance, comprehensibility, and clarity of items. We asked patients whether the MDASI-MM encompassed major symptoms of concern; if the items presented were relevant and if any that seemed important were missing; if the questions were easy to understand; if any items seemed redundant; whether the 0–10 numeric scale was easy to use; whether patients were comfortable answering the questions; and if they had suggestions for making the questions more comfortable to answer. The final MDASI-MM contains the MDASI’s 13 core symptom severity items and 6 interference items [8], plus 7 MM-specific items (bone aches, muscle weakness, sore mouth/throat, rash, difficulty concentrating, constipation, diarrhea). Patients rate symptoms on a 0–10 scale ranging from “not present” to “as bad as you can imagine.” Interference is rated on a 0–10 scale ranging from “did not interfere” to “interfered completely.”

#### Scoring

MDASI-MM ratings can be used to derive 3 subscale scores: mean *core* (13 MDASI core symptom items), mean *severity* (13 MDASI core plus 7 MM-specific items), and mean *interference* (6 interference items). The interference items can be subdivided into mean activity-related (interference with work, general activity, and walking ability (WAW)) and mean mood-related (interference with relations with people, enjoyment of life, and mood (REM)) dimensions [17]. Symptom severity can be classified as 0 (none), 1–4 (mild), 5–6 (moderate), or 7–10 (severe) [17,18].

### Criterion measures

#### EORTC QLQ-C30 and QLQ-MY20

The QLQ-C30 is a reliable, valid 30-item instrument used to measure QOL in cancer patients [19]. The QLQ-MY20 is a reliable, valid 20-item measure used in combination with the QLQ-C30 to assess QOL in MM patients [7]. Both consist of functional and symptom/side-effect subscales and items. Higher functional subscale scores represent higher levels of functioning, whereas higher symptom/side-effect subscale and item scores represent more-severe symptoms/side effects.

#### Eastern Cooperative Oncology Group performance status

Eastern Cooperative Oncology Group performance status (ECOG PS) is used to estimate patients’ functional status and to determine appropriate treatment and prognosis [20]. ECOG PS is a reliable, valid 5-point measure ranging from fully active (0) to deceased (5) [21,22].

### Statistical analyses

Analyses were conducted using Statistical Package for the Social Sciences version 17.0 (SPSS Inc., Chicago, IL). Descriptive statistics were computed for symptoms and subscales. Statistical significance was set at a 2-tailed α level of .05.

#### Reliability

Internal consistency reliability of the MDASI-MM was estimated by calculating Cronbach α values for the MDASI-MM core, severity, interference subscales at baseline (first MDASI-MM assessment). A Cronbach α value ≥0.70 indicates good internal consistency reliability [23].

#### Criterion validity

Criterion validity was examined by correlating selected MDASI-MM items and subscales with those from the QLQ-C30 and QLQ-MY20 at baseline. We hypothesized that the MDASI-MM’s activity-related and mood-related interference subscales would correlate with the physical-function and emotional-function subscales, respectively, of the QLQ-C30, and that the MDASI-MM’s symptom subscales would correlate with the symptom/side-effect subscales of the QLQ-MY20. Because the QLQ-MY20 disease-symptoms subscale contains primarily pain and bone-ache-related items [7], we examined correlations of the individual MDASI-MM “pain” and “bone aches” items with this subscale.

#### Construct validity

Construct validity of the MDASI-MM was assessed by evaluating its ability to distinguish between groups of patients with clinical differences in ECOG PS (ie, good (≤1) versus poor (≥2)) after induction or transplant.

#### Sensitivity

To evaluate the sensitivity of the MDASI-MM, we used 2 methods.

First, we assessed the MDASI-MM’s ability to detect change in ECOG PS during the course of treatment. We compared change in MDASI-MM ratings with change in ECOG PS scores between 2 time points: either between the first and last MDASI-MM assessments during induction therapy or between pre-HSCT and 7 days post-HSCT. ECOG PS was considered stable/improving if it remained the same or decreased relative to baseline, and worsening if it increased one or more points during treatment. Change scores for the MDASI-MM subscales and individual items were considered clinically meaningful at 0.5 standard deviation (SD) or higher [24]. We anticipated that the stable/improving group would have no significant change in MDASI-MM ratings but that the worsening group would; therefore, we conducted 1-sample *t*-tests to examine change in ratings for each MDASI-MM subscale.

Second, we examined the sensitivity of the MDASI-MM to detect the acute worsening of the transplant sample’s symptoms post-HSCT. We used paired *t*-tests to examine change in scores for the MDASI-MM subscales and individual items. Effect sizes were calculated and an effect size >0.5 was considered clinically meaningful [25,26].

## Results

Demographic and clinical characteristics of the samples are summarized in Table 1. Mean age for the induction sample (n = 64) was 62.9 years and for the transplant sample (n = 68) was 62.1 years. Patients in both samples were predominantly White non-Hispanic and had good (0–1) baseline ECOG PS.

**Table 1:** Demographic and clinical characteristics

	**Induction**		**Transplant**	
	**(*n*= 64)**		**(*n*= 68)**	
**Patient characteristics, mean (SD)**				
Age, years	62.9	(11.98)	62.1	(7.42)
Education level, years	14.8	(2.20)	14.5	(2.18)
**Patient characteristics, % (no.)**				
Sex				
Men	59.4%	(38)	69.1%	(47)
Women	40.6%	(26)	30.9%	(21)
Race and ethnicity				
White non-Hispanic	73.4%	(47)	77.9%	(53)
Hispanic	6.3%	(4)	10.3%	(7)
Black	12.5%	(8)	10.3%	(7)
Other	7.8%	(5)	1.5%	(1)
Baseline ECOG PS				
Good (0–1)	82.8%	(53)	95.5%	(64)
Poor (2+)	17.2%	(11)	4.5%	(3)

### Validation of the MDASI-MM

#### Internal consistency reliability

The MDASI-MM subscales showed good reliability. Cronbach coefficient α values were 0.85 for the core subscale, 0.88 for the severity subscale, and 0.91 for the interference subscale.

#### Criterion validity

The MDASI-MM’s severity subscale displayed good association with the QLQ-C30 physical, role, cognitive, social, and emotional-functioning subscales (all *P* < .001) (Table 2). As hypothesized, the MDASI-MM’s activity-related interference subscale correlated more strongly with the QLQ-C30 physical-function subscale, whereas its mood-related-interference subscale correlated more strongly with the emotional-function subscale (all *P* < .001). MDASI-MM symptom subscales correlated well with the disease-symptoms and side-effects subscales of the QLQ-MY20 (all *P* < .001).

**Table 2:** **Criterion validity: MDASI-MM subscales correlated with QLQ-C30 and QLQ-MY20 subscales (*n*= 55)**

**MDASI-MM**	**QLQ-C30**					**QLQ-MY20**	
	**Physical function**	**Role function**	**Emotional function**	**Cognitive function**	**Social function**	**Disease symptom**	**Side effect**
Core	−0.43^a^	−0.37^a^	−0.60^b^	−0.66^b^	−0.41^a^	0.54^b^	0.67^b^
Severity	−0.49^b^	−0.44^b^	−0.60^b^	−0.69^b^	−0.47^b^	0.60^b^	0.68^b^
Interference	−0.70^b^	−0.70^b^	−0.51^b^	−0.49^b^	−0.64^b^	0.70^b^	0.49^b^
WAW	−0.74^b^	−0.72^b^	−0.39^a^	−0.43^a^	−0.60^b^	0.70^b^	0.44^b^
REM	−0.56^b^	−0.57^b^	−0.60^b^	−0.50^b^	−0.61^b^	0.60^b^	0.49^b^

^a^ Significant at *P* < .05.

^b^ Significant at *P* < .001.

Individual MDASI-MM symptom items correlated strongly with comparable items and relevant subscales of the QLQ-C30 and QLQ-MY20 (Table 3). Strong correlations existed for pain, fatigue, nausea, shortness of breath, difficulty remembering, lack of appetite, dry mouth, numbness, constipation, diarrhea, and bone aches (all *P* < .001). Correlations between the MDASI-MM pain and bone-aches items and the QLQ-MY20 disease-symptoms subscale were 0.71 and 0.69 (*P* <.001), respectively.

**Table 3:** **Criterion validity: MDASI-MM items compared with individual items or relevant subscales from the QLQ-C30 and QLQ-MY20 (*n*= 55)**

**MDASI-MM individual symptom items**	**QLQ C-30 and QLQ MY-20 question number(s)**^**a**^**(subscale)**	**Pearson’s correlation**^**b**^
Pain	Q-9, 19 (QLQ C-30 pain subscale)	0.76^c^
Fatigue	Q-10, 12, 18 (QLQ C-30 fatigue subscale)	0.75^c^
Nausea	Q-14	0.77^c^
Shortness of breath	Q-8	0.67^c^
Difficulty remembering	Q-25	0.75^c^
Lack of appetite	Q-13	0.66^c^
Dry mouth	Q-40	0.86^c^
Numbness	Q-43	0.66^c^
Constipation	Q-16	0.75^c^
Diarrhea	Q-17	0.71^c^
Bone aches	Q-31	0.69^c^

^a^ Numbered questions: Q-9: Have you had pain?; Q-19: Did pain interfere with your daily activities?; Q-10: Did you need to rest?; Q-12: Have you felt weak?; Q-18: Were you tired?; Q-14: Have you felt nauseated?; Q-8: Were you short of breath?; Q-25: Have you had difficulty remembering things?; Q-13: Have you lacked appetite?; Q-40: Have you had a dry mouth; Q-43: Did you have tingling hands or feet?; Q-16: Have you been constipated?; Q-17: Have you had diarrhea?; Q-31: Have you had bone aches or pain?

^b^ Spearman’s correlations demonstrated similar magnitude as Pearson’s correlations.

^c^ Significant at *P* < .001.

#### Construct (known-group) validity

Patients with good ECOG PS had significantly lower scores for the MDASI-MM subscales than patients with poor ECOG PS (all *P* < .001) (Table 4). Similar results were seen for the MM-specific items (*P* < .001). Effect sizes for the differences were ≥0.7, indicating medium to large effects.

**Table 4:** **Known-group validity: MDASI-MM symptom and interference subscale scores compared with ECOG PS scores from final MDASI-MM assessment (*n*= 127)**

**MDASI-MM subscale**	**ECOG PS**	**No. of patients**	**Mean**	**SD**	**Difference**	**95% CL**		**Effect size**
						**Lower**	**Upper**	
Core	Good	63	1.88	1.50	1.62^a^	1.50	2.26	0.89
	Poor	64	3.50	1.79		3.06	3.95	
Severity	Good	63	1.72	1.38	1.43^a^	1.37	2.07	0.86
	Poor	64	3.15	1.60		2.75	3.55	
Interference	Good	63	2.71	2.14	1.91^a^	2.17	3.25	0.75
	Poor	64	4.62	2.57		3.98	5.26	

^a^ Significant at *P* < .001.

#### Sensitivity to change in performance status

MDASI-MM subscale change scores were significant for patients with worsening ECOG PS (core: t(65) = 8.350; severity: t(65) = 8.672; interference: t(65) =7.344; all *P* < .001), but were not significant for patients with stable/improving ECOG PS. Difference scores between patients whose ECOG PS worsened and patients for whom it remained stable/improved were statistically significant for each MDASI-MM subscale (all *P* < .001, all effect sizes ≥0.7) (Table 5). Additionally, difference scores between the groups were statistically significant for most individual symptom items.

**Table 5:** **Sensitivity of the MDASI-MM to change in ECOG PS (*n*= 126)**

	**Stable/improving ECOG PS (*n*= 60)**		**Worsening ECOG PS (*n*= 66)**		**Difference**	**Effect size**
	**Mean**^**a**^	**SD**	**Mean**^**a**^	**SD**		
Change in composite scores						
Core subscale	0.07	1.54	1.66	1.62	1.59^b^	0.90
Severity subscale	0.09	1.35	1.52	1.42	1.43^b^	0.91
Interference subscale	0.16	2.63	2.15	2.38	1.99^b^	0.74
Change in MDASI-MM items						
Dry mouth	−0.92	3.49	3.44	3.33	4.36^b^	1.08
Fatigue	−0.28	3.11	2.44	2.68	2.72^b^	0.85
Lack of appetite	0.70	3.09	3.85	3.62	3.15^b^	0.85
Nausea	0.68	2.25	3.48	3.60	2.80^b^	0.84
Drowsiness	−0.52	3.92	2.23	2.92	2.75^b^	0.74
Diarrhea	1.15	3.01	3.68	3.67	2.53^b^	0.71
Vomiting	0.40	1.43	1.89	3.10	1.49^c^	0.59
Mouth/throat sores	0.37	2.18	1.86	3.41	1.49^c^	0.50
Pain	−0.48	3.35	1.15	3.09	1.63^c^	0.49
Muscle weakness	0.42	3.29	1.83	2.52	1.41^c^	0.47
Numbness	0.90	2.70	−0.24	2.21	−1.14^c^	0.45
Disturbed sleep	0.02	3.41	1.20	3.21	1.18^c^	0.35
Bone aches	−1.03	3.59	0.13	3.17	1.16^c^	0.34
Shortness of breath	−0.08	2.23	0.68	2.23	0.76	0.34
Sadness	−0.02	2.00	0.55	2.77	0.57	0.23
Difficulty paying attention	0.25	1.87	0.65	2.12	0.40	0.20
Difficulty remembering	0.02	1.78	0.27	1.62	0.25	0.15
Constipation	−0.50	2.94	−0.20	2.24	0.30	0.11
Distress	0.52	2.66	0.68	2.97	0.16	0.06
Rash	0.32	1.58	0.34	2.16	0.02	0.01

^a^ A positive mean indicates increased symptom intensity; negative indicates decreased symptom intensity.

^b^ Significant at *P* < .001.

^c^ Significant at *P* < .05.

#### Sensitivity to impact of therapy (HSCT)

Substantial and statistically significant increases in patients’ scores between pre-HSCT and 7 days post-HSCT were observed, with effect sizes of 1.29, 1.33, and 0.95 for the core, severity, and interference subscales, respectively (all *P* < .001) (Table 6). Differences in individual symptoms were also clinically significant for most items, with effect sizes ≥0.5.

**Table 6:** **Sensitivity of the MDASI-MM to impact of therapy: comparison of subscale and individual symptom scores pre-HSCT and 7 days post-HSCT (*n*= 66)**

	**Pre-HSCT**		**Post-HSCT**		**Difference**^**a**^	**Effect size**
	**Mean**	**SD**	**Mean**	**SD**		
MDASI-MM subscales						
Core	1.60	1.30	3.57	1.76	1.97^b^	1.29
Severity	1.43	1.14	3.21	1.62	1.78^b^	1.33
Interference	2.32	2.29	4.51	2.62	2.19^b^	0.95
Individual items						
Lack of appetite	0.96	1.77	5.73	3.14	4.77^b^	1.43
Diarrhea	0.56	1.25	5.37	3.39	4.81^b^	1.36
Nausea	0.36	0.96	4.38	3.28	4.02^b^	1.20
Dry mouth	1.22	2.14	4.94	3.35	3.72^b^	1.11
Fatigue	2.99	2.52	5.86	2.40	2.87^b^	1.07
Drowsiness	1.88	2.12	4.53	2.56	2.65^b^	0.88
Mouth/throat sores	0.34	0.99	2.82	3.29	2.48^b^	0.81
Vomiting	0.06	0.29	2.29	3.16	2.23^b^	0.70
Muscle weakness	1.92	2.10	3.45	2.37	1.53^b^	0.56
Disturbed sleep	2.47	2.61	4.03	2.79	1.56^b^	0.49
Distress	1.82	2.41	3.03	2.91	1.21^b^	0.48
Pain	2.84	2.80	4.11	2.98	1.27^c^	0.44
Sadness	1.37	2.34	2.05	2.92	0.68^c^	0.30
Difficulty paying attention	1.06	1.57	1.57	2.01	0.51^c^	0.29
Shortness of breath	0.81	1.54	1.39	2.12	0.58^c^	0.25
Rash	0.38	1.33	0.88	2.00	0.50	0.21
Constipation	0.95	1.67	0.65	1.96	−0.30	0.20
Difficulty remembering	1.21	1.90	1.44	1.83	0.23	0.15
Numbness	2.90	3.00	2.70	2.99	−0.20	0.09
Bone aches	2.31	2.38	2.48	2.76	0.17	0.03

^a^ Positive values indicate mean increase in symptoms post-HSCT; negative indicate mean symptom improvement.

^b^ Significant at *P* < .001.

^c^ Significant at *P* < .05.

### Cognitive debriefing

Cognitive debriefing interviews were conducted with 20 patients. All reported that they were comfortable answering the MDASI-MM questions; that the questionnaire was easy to understand, and that it was comprehensive and non-repetitive. One patient reported swelling/edema and night sweats. No other MM or treatment-related symptoms were suggested during open interviews.

### Symptom severity

In order of severity, the most-severe symptoms reported at induction baseline were fatigue, pain, disturbed sleep, drowsiness, bone aches, dry mouth, and muscle weakness; 35.9% of patients reported moderate to severe pain (≥5 on the MDASI-MM) and 45.3% reported moderate to severe fatigue [17]. At the end of induction, the most-severe symptoms reported were fatigue, pain, muscle weakness, disturbed sleep, drowsiness, numbness, and bone aches; 27.4% of patients reported moderate to severe pain and 30.6% reported moderate to severe fatigue.

**Figure 1:**
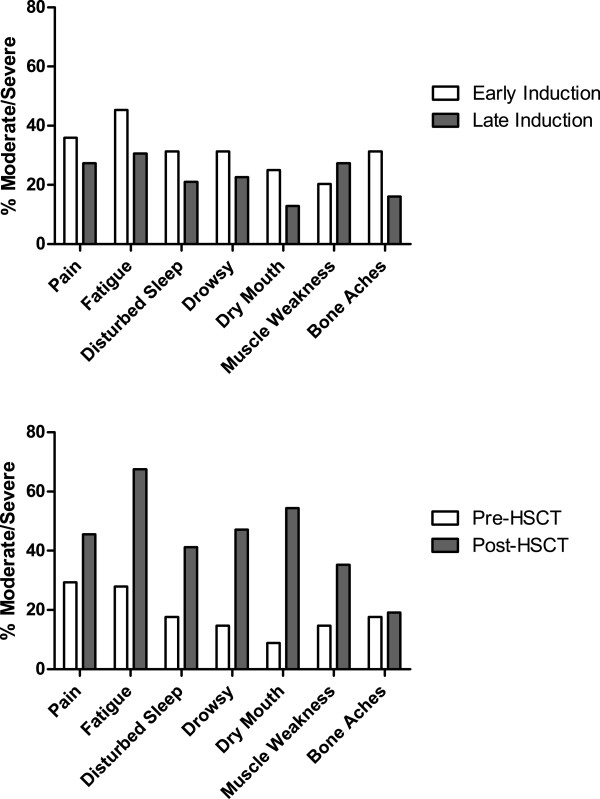
Comparison of symptom severity for top 7 symptoms during induction chemotherapy with their levels before and 7 days after hematopoietic stem cell transplant.

The most-severe symptoms reported pre-HSCT were fatigue, numbness, pain, disturbed sleep, bone aches, and muscle weakness; 29.4% of patients reported moderate to severe pain, and 27.9% reported moderate to severe fatigue. Post-HSCT scores reflected a significant increase in symptom burden. The most-severe symptoms reported post-HSCT were fatigue, lack of appetite, diarrhea, dry mouth, drowsiness, nausea, and pain (Figure 1).

#### MDASI-MM versus EORTC instruments

To examine how the top symptoms captured by the MDASI-MM would compare with the top symptoms captured by the QLQ C-30 and QLQ-MY20, we excluded non-symptom items from the latter scales and compared the top 5 symptoms identified by each instrument. Although in different order of severity, 4 of the top 5 symptoms captured by the MDASI-MM (fatigue, pain, disturbed sleep, drowsiness, bone aches) were identical to those captured by the 2 EORTC instruments (bone aches, fatigue, pain, disturbed sleep, and worry about health).

## Discussion

This study evaluated an MM-specific module of the MDASI in patients undergoing treatment for MM. Our results provide strong psychometric evidence that the MDASI-MM is a valid, reliable instrument that can discriminate between clinically different patient groups and that has high sensitivity in detecting symptom change during treatment with respect both to patients’ ECOG PS and evolving symptom trajectories. Individual items and subscales of the MDASI-MM correlated strongly with corresponding items and subscales of the EORTC QLQ C-30 and QLQ MY-20. Cognitive debriefing evidenced that the MDASI-MM is easy to understand, quick to complete, and comprehensive.

One of the most useful assets of the MDASI-MM is its brevity. For example, it captured 4 of the top 5 symptoms captured by the 2 EORTC instruments. Thus, although brief, it captures patients’ most critical symptoms with minimal patient burden. Additionally, unlike the MDASI-MM, the 2 EORTC instruments are QOL scales that were not developed within the conceptual framework of symptom assessment [7,19]. As the QLQ C-30 must be administered with the QLQ MY-20 (a total of 50 items, compared with 26 items in the MDASI-MM), this adds substantially to patient burden. The MDASI-MM’s brevity also allows for efficient repeat administrations for charting a patient’s symptom trajectory over the course of treatment, which is a valuable aid to clinical decision making. Previous research indicates that patient-reported outcome measures can be effective guides to key clinical decisions [27], especially when objective evaluations of physical indices are difficult [28].

A significant advantage of MDASI modules is that each module includes all symptom and interference items from the original MDASI. Each module validation subjects the original MDASI items to further cognitive debriefing and sensitivity testing by additional patients, reinforcing the validity, reliability, and sensitivity of the original instrument. Original items can thus be included in new modules with fewer psychometric steps, enabling efficiency and cost savings in the development of disease-specific modules. Also, because each new module includes the original MDASI items, the use of MDASI modules facilitates comparison of symptom prevalence and severity across cancer types, a significant benefit in clinical trials or studies that include patients with different cancers.

During cognitive debriefing, 1 patient reported 2 symptoms not present in the MDASI-MM module (swelling/edema and transient night sweats). These items were not included in the final instrument as they were extremely rare [16]. If these items are reported in the literature with greater frequency, it may be reasonable to include them in a future revision of the MDASI-MM.

A limitation of our study was its largely racially homogenous population; future work is needed to test patients of diverse backgrounds in various treatment settings. Another limitation is that although we were able to demonstrate the sensitivity of the MDASI-MM to change in performance status and to impact of therapy, we were unable to demonstrate its sensitivity to change in patients’ disease status (eg, stable or progressive disease; partial or very good partial response) as these data were not available. Our key strengths include the development of an instrument based on symptom burden rather than health-related QOL, and inclusion of both induction and transplant patients, which extends its generalizability.

## Conclusions

Numerous clinical trials are underway for patients with MM. Having a uniform, validated assessment instrument to monitor disease-related and treatment-related symptoms is therefore essential. Our study establishes that the MDASI-MM is a reliable, valid instrument developed with both patient and clinician input. Additionally, due to its brevity, simple 0–10 assessment scale, and ease of administration, it is a universally practical tool. The original MDASI is available in multiple linguistically and psychometrically validated versions [29-33], and MDASI modules such as the MDASI-MM are readily translatable. We recommend the MDASI-MM as a uniform symptom assessment instrument for patients with MM in treatment settings and clinical trials.

## Abbreviations

ECOG PS: Eastern cooperative oncology group performance status;EORTC: European Organisation for Research and Treatment of Cancer;HSCT: Hematopoietic stem cell transplant;MDASI: M. D. Anderson Symptom Inventory;MDASI-MM: M. D. Anderson Symptom Inventory multiple myeloma module;MM: Multiple myeloma;QLQ-C30: EORTC Quality of Life Questionnaire;QLQ-MY20: EORTC Multiple Myeloma Module;QOL: Quality of life;REM: Mood-related interference with functioning, including relations with people, enjoyment of life, and mood;WAW: Activity-related interference with functioning, including symptom interference with work, general activity, and walking ability

## Competing interests

The authors declare that they have no competing interests.

## Authors’ contributions

DJ conducted patient interviews (cognitive debriefing), participated in data interpretation, and wrote the manuscript; EGV and TRM conducted statistical analysis, contributed to data interpretation, and reviewed the manuscript; XSW, LAW, and CSC designed the research study, participated in performing the research, and reviewed the manuscript; VEJ contributed to the statistical design and reviewed the manuscript., NDS, SKT, and REC contributed to the research design, performed patient recruitment, and reviewed the manuscript. All authors read and approved the final manuscript.

## Statement of Prior Presentation

Presented at the 48th Annual Meeting of the American Society of Clinical Oncology, Chicago, IL, June 1-5, 2012.

## Supplementary Material

Additional file 1: Figure S1 The MDASI-MM. Click here for file
