# Supplementary material for: Validation of the M. D. Anderson Symptom Inventory multiple myeloma module
Source: J Hematol Oncol. 2013 Feb 5;6:13. doi: 10.1186/1756-8722-6-13 (PMC3598689; doi:10.1186/1756-8722-6-13)
Supplement: Additional file 1: Figure S1 — The MDASI-MM. [file 1756-8722-6-13-S1.pdf]

Date: ______________________ Institution:______________________

Participant Initials: ______________ Hospital Chart #:__________________

Participant Number: __________________

M. D. Anderson Symptom Inventory - Multiple Myeloma (MDASI - MM)

Part I. How **severe** are your symptoms?

People with cancer frequently have symptoms that are caused by their disease or by their treatment. We ask you to rate how severe the following symptoms have been ***in the last 24 hours.*** Please fill in the circle below from 0 (symptom has not been present) to 10 (the symptom was as bad as you can imagine it could be) for each item.

	Not Present 0	1	2	3	4	5	6	7	8	9	As Bad As You Can Imagine 10
1. Your **pain** at its WORST?	○	○	○	○	○	○	○	○	○	○	○
2. Your **fatigue (tiredness)** at its WORST?	○	○	○	○	○	○	○	○	○	○	○
3. Your **nausea** at its WORST?	○	○	○	○	○	○	○	○	○	○	○
4. Your **disturbed sleep** at its WORST?	○	○	○	○	○	○	○	○	○	○	○
5. Your feelings of being **distressed (upset)** at its WORST?	○	○	○	○	○	○	○	○	○	○	○
6. Your **shortness of breath** at its WORST?	○	○	○	○	○	○	○	○	○	○	○
7. Your problem with **remembering** things at its WORST?	○	○	○	○	○	○	○	○	○	○	○
8. Your problem with **lack of appetite** at its WORST?	○	○	○	○	○	○	○	○	○	○	○
9. Your feeling **drowsy (sleepy)** at its WORST?	○	○	○	○	○	○	○	○	○	○	○
10. Your having a **dry mouth** at its WORST?	○	○	○	○	○	○	○	○	○	○	○
11. Your feeling **sad** at its WORST?	○	○	○	○	○	○	○	○	○	○	○
12. Your **vomiting** at its WORST?	○	○	○	○	○	○	○	○	○	○	○
13. Your **numbness or tingling** at its WORST?	○	○	○	○	○	○	○	○	○	○	○

Copyright 2000 The University of Texas M. D. Anderson Cancer Center
All rights reserved.

Date: ____________________________ **Institution:**____________________________

Participant Initials: __________________ **Hospital Chart #:**______________________

Participant Number: ______________________

**MM**	Not Present 0	1	2	3	4	5	6	7	8	9	As Bad As You Can Imagine 10
14. Your **constipation** at its WORST?	○	○	○	○	○	○	○	○	○	○	○
15. Your **muscle weakness** at its WORST?	○	○	○	○	○	○	○	○	○	○	○
16. Your **diarrhea (loose stools)** at its WORST?	○	○	○	○	○	○	○	○	○	○	○
17. Your **sore mouth or throat** at its WORST?	○	○	○	○	○	○	○	○	○	○	○
18. Your **rash** at its WORST?	○	○	○	○	○	○	○	○	○	○	○
19. Your problem with **Paying Attention (Concentrating)** at its WORST?	○	○	○	○	○	○	○	○	○	○	○
20. Your **bone aches** at its WORST?	○	○	○	○	○	○	○	○	○	○	○

Part II. How have your symptoms interfered with your life?

Symptoms frequently interfere with how we feel and function. How much have your symptoms interfered with the following items ***in the last 24 hours***:

	Did Not Interfere 0	1	2	3	4	5	6	7	8	9	Interfered Completely 10
21. **General activity?**	○	○	○	○	○	○	○	○	○	○	○
22. **Mood?**	○	○	○	○	○	○	○	○	○	○	○
23. **Work** (including work around the house)?	○	○	○	○	○	○	○	○	○	○	○
24. **Relations** with other people?	○	○	○	○	○	○	○	○	○	○	○
25. **Walking?**	○	○	○	○	○	○	○	○	○	○	○
26. **Enjoyment of life?**	○	○	○	○	○	○	○	○	○	○	○

Copyright 2000 The University of Texas M. D. Anderson Cancer Center
All rights reserved.
